# Personality traits, workers’ age, and job satisfaction: The moderated effect of conscientiousness

**DOI:** 10.1371/journal.pone.0252275

**Published:** 2021-07-26

**Authors:** Eleonora Topino, Annamaria Di Fabio, Letizia Palazzeschi, Alessio Gori

**Affiliations:** 1 Department of Human Sciences, LUMSA University of Rome, Rome, Italy; 2 Department of Education, Languages, Interculture, Letters and Psychology (Psychology Section), University of Florence, Florence, Italy; 3 Department of Health Sciences–University of Florence, Florence, Italy; Universitat de Valencia, SPAIN

## Abstract

Job satisfaction has gained increasing interest in the world of work and a vast field of research has been stimulated regarding its antecedents. Among these, personality traits have received consistent and significant attention, with a particular emphasis on conscientiousness. To delve deeper and detail these aspects, in the present research, a moderation model was hypothesized, with the aim of investigating the effect of age on the association between conscientiousness (and its subdimensions scrupulousness and perseverance) and job satisfaction. The age-moderated interactions of the other Big Five personality traits were also explored. The study involved 202 Italian workers (92 men, 110 women) with a mean age of 44.82 years (*SD* = 10.56) who completed the Big Five Questionnaire and the Job Satisfaction Scale. The results showed a positive association between conscientiousness and job satisfaction. This was moderated by age to the extent that it was significant for younger and average-age workers and was less significant for older workers. Similar results were found for the subdomain of perseverance, while the relationship between scrupulousness and job satisfaction was not significant. Furthermore, no age-moderated interaction between the other Big Five personality traits and Job satisfaction were found. Such data supports interactive models that highlight the need to integrate personality traits with other factors in exploring the antecedents of job satisfaction. These findings provide additional elements to an understanding of the factors contributing to workers satisfaction, and could have important applicative implications in a framework for healthy organizations and the well-being movement.

## Introduction

Job satisfaction is a construct that is increasingly growing and attracting consistent interest in the field of work and organizational psychology [1]. It includes cognitive, affective, and behavioral aspects [2] and can be defined as “*an evaluative state that expresses contentment with*, *and positive feelings about*, *one’s job*” [3] (p. 347).

Research into job satisfaction has focused, above all, on the consequences resulting from different levels of job satisfaction and on the antecedents of this construct. Job satisfaction is seen as having numerous applications and repercussions both at work and on people’s everyday lives [2,3]. Job satisfaction is shown to be associated with numerous organizational outcomes, such as higher commitment [4], greater job involvement [5], improved performance [6], better organizational citizenship [7], and also with lower levels of turnover intentions [8], less absenteeism [9] and fewer counterproductive work behaviors [10,11]. Moreover, job satisfaction can be considered a sub-domain of the larger construct, life-satisfaction, which in turn, is a component of subjective well-being [12–14]. Previous research has reported negative relationships between job satisfaction and distress [15], burnout [16,17], and anxiety [18], as well as positive associations between job satisfaction and marital satisfaction [19,20], happiness [21], and psychophysical health [18,22]. Therefore, evidence supports the importance of focusing on job satisfaction both for its effect on organizational functioning [23] and for ethical reasons: according to the vision for decent work [24–27], work should be sustainable and meaningful within a broader framework of a decent life [28,29]. Job satisfaction is an essential construct in the framework of healthy organizations [30,31]. This perspective underlines the relevance to explore personality and individual differences in relation to workers’ well-being. Job satisfaction also emerged as important in the framework of the well-being movement [32,33] that highlights the value of reducing negative outcomes and promoting individual resources to enhance both well-being and productivity. This supports both the relevance of workers’ well-being to organizational success and healthy business, and its critical link with strength-based prevention perspectives [34]. Therefore, the well-being of employees and organizational performance are both at the center of focus and are nourished by one another [33,35], where health is seen as optimal functioning [36–38] and a reflection of one’s satisfaction with both work and life [39,40].

Given this evidence concerning its applicability and centrality to the working world, a study of the precursors to satisfaction with one’s work appears both necessary and useful [3,41,42]. In support of this perspective, the general aim of the present research was to deepen the understanding of the antecedents to job satisfaction.

The scientific literature concerning the variables associated with job satisfaction highlights several approaches, which can be grouped into dispositional, environmental, and integrative [2]. With regards to individual factors, personality traits emerge as important predictors of job satisfaction [43,44], with particular reference to the Big Five model of Costa and McCrae [45], which has proved particularly effective in the study of the dispositional sources of job satisfaction [44]. This structure does not imply that personality differences can be reduced to just five traits (extraversion, agreeableness, conscientiousness, emotional stability, and openness), but with these, it is possible to obtain a sufficiently complete and exhaustive representation, in which each dimension summarizes and contains other, more specific, characteristics [45,46]. Among the five traits, the conscientiousness one (consisting in the dimensions of scrupulousness and perseverance) [45,47] has been showed as particularly relevant in the organizational context by several studies, highlighting its associations with important work outcomes, such as attitudes [48,49], job satisfaction [50], performance [51,52], relationships with leaders [53], response to workplace stressors [54], and organizational citizenship behaviors [55]. More specifically, the relationship with job satisfaction was also pointed by previous meta-analyzes, which showed that conscientiousness showed significant positive associations [13], sometimes the highest among different traits of the big five [44,56,57]. Conscientious people tend to be well-organized, self-disciplined, hardworking, growth and success-oriented, persevering, and motivated in the pursuit of established goals [58,59]. Taken together, these factors favor greater efficiency and increase the probability of obtaining satisfactory rewards in the work context, both formally (e.g., promotion) and informally (e.g., esteem and reputation) [44].

However, although the scientific literature has highlighted the association between personality traits and job satisfaction [44], these dispositions may not play an exclusive role in determining motivation and job satisfaction during working life [60]. For example, the Baltes’ model of Selective Optimization with Compensation (SOC) [61] argues that old age is associated with a decline in motivation concerning job growth and an increase in factors relating to regulation-loss and the maintenance of status. Therefore, the energy invested in the initial objectives of success and the expenditure of effort toward achievement that is typical of the younger worker tends to be replaced by a reallocation of resources toward preserving one’s own situation and avoiding losses in older age [62,63].

**Fig 1:**
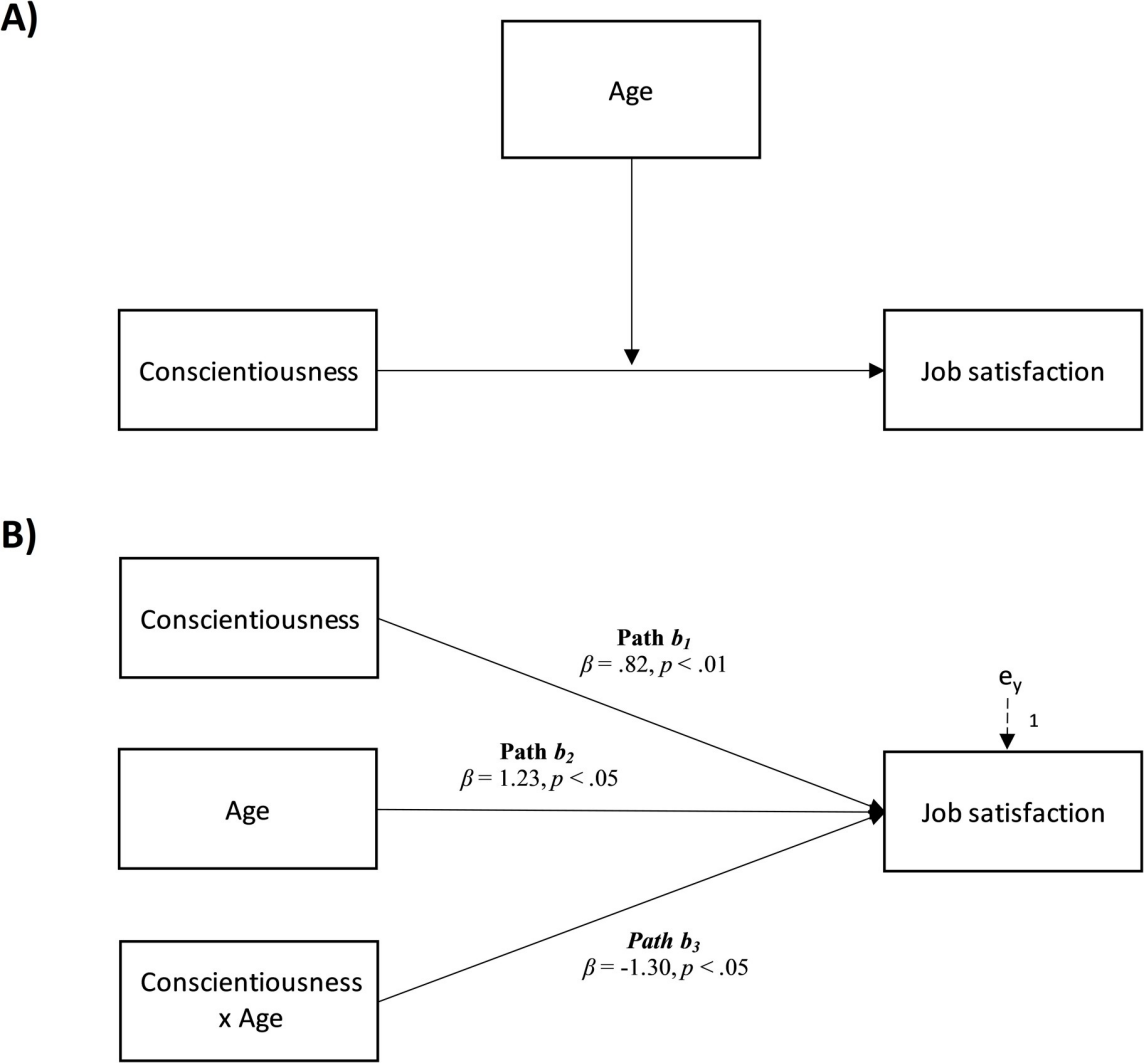
Conceptual (A) and statistical (B) models. The moderation of age on the relationship between Conscientiousness and Job satisfaction.

Based on this theoretical framework, the purpose of this study is to gain insights regarding the relationships between workers’ ages, conscientiousness, and job satisfaction. Therefore, a moderation model was hypothesized, in which the interaction between conscientiousness and job satisfaction was moderated by age (see Fig 1A), with the expectation that the association with this personality trait is stronger for younger workers. Furthermore, the age-moderated relationship between the subdomains of conscientiousness (scrupulousness and perseverance) and job satisfaction was explored.

## Method

### Participants and procedure

The study involved a sample of 202 Italian workers (45.5% men, 54.5% women), with a mean age of 44.82 years (*SD* = 10.56, age range 25–64 years). They were recruited from various private Tuscan organizations, and their participation in this study was voluntary. All respondents completed a paper and pencil self-report questionnaire administered by the researchers, without receiving any form of compensation, and they were free to leave the study at any time. Furthermore, participants were informed about the general aim of the research and were asked to complete a written informed consent form before starting. This protocol had been approved by the Ethical Committee of the Integrated Psychodynamic Psychotherapy Institute (IPPI).

### Measures

#### Big Five Questionnaire (BFQ)

The Big Five Questionnaire (BFQ) [47] is a self-report measure for the assessment of personality in line with the Costa and McCrae Big Five Theory [45]. Caprara et al. [64] reported good psychometric properties and satisfactory internal consistency in the original study. It consists of 132 items answered on a five-point Likert scale (from “*Absolutely false*” to “*Absolutely True*”). These are grouped into five dimensions with an additional scale indicating truthfulness: (1) Extraversion (24 items; α = .81) includes the Dynamism and Dominance subdimensions and indicates a confident and enthusiastic tendency toward the various circumstances of life; (2) Agreeableness (24 items; α = .73) includes the Cooperativeness and Politeness subscales and indicates a tendency to be empathetic and cooperative (or suspicious and hostile); (3) Conscientiousness (24 items; α = .81) includes the Scrupulousness and Perseverance subdimensions and indicates a tendency to be organized, precise, self-disciplined, dependable, and persevering, with a preference for planned rather than spontaneous activities; (4) Emotional Stability (24 items; α = .90) includes the Control of the emotions and Control of the impulses subscales and indicates the degree of emotional stability and impulse control; (5) Openness (24 items; α = .75) comprises the Openness to culture and Openness to experience subscales and indicates a tendency to be open toward new ideas, other’s values, and one’s own feelings; (6) Lie (12 items; α = .74), antruthfulness indicator which concerns the subject’s tendency to provide a distorted profile, whether positively or negatively.

#### Job Satisfaction Scale (JSS)

The Job Satisfaction Scale (JSS) [65] is a self-report questionnaire used to assess a worker’s satisfaction with their own job. It consists of five items answered on a seven-point Likert scale (from “*strongly disagree*” to “*strongly agree*”). The Italian version of Di Fabio [66] was used in this study, which showed good internal consistency (α = .89).

### Data analysis

The SPSS software (v. 25.0 for Windows) was used to analyze the collected data. Descriptive statistics were examined for the sample and the measures. Pearson’s *r* correlations and Partial correlation controlling for age and gender were calculated to explore the association between variables. Furthermore, the hypothesized moderation model and the explorative ones involving the subdimensions of conscientiousness (scrupulousness and perseverance) were investigated by using the macro-program PROCESS v. 3.4 [67] and applying Model 1. The moderated effects were tested by performing the bootstrapping technique with 95% confidence intervals (CI) with 5000 samples and then by using the Wayne et al. [68] procedure. Concerning the first, a bootstrapped confidence interval (from “LLCI = Lower Limit” to “ULCI = Upper Limit”) not including zero indicates the significance of the effect. For the second, following Wayne et al. [68], the conditional effect was tested by analyzing the index of the moderated relationship (and confidence intervals) for “low,” “average,” and “high” levels of the moderator (-1DS, Mean, +1DS), considering a *p* level of < .05 to define statistical significance. Post-hoc power analyses were conducted to assess the achieved power for the moderation analyses given a sample size of 202 and an alpha of .05, by using G*Power 3 software [69] for Linear multiple regression (Fixed model, *R^2^* deviation from zero): a power of at least 0.80 is considered as the recommended value for the social sciences [70,71]. Similarly, alternative models were also tested, to explore relationship between different Big Five personality dimensions and job satisfaction, with the moderation of age.

## Results

The correlation analyses and descriptive statistics are shown in Table 1.

**Table 1:** Pearson’s correlation matrix (below the diagonal), Partial correlation controlling for Gender and Age (above the diagonal), and descriptive statistics.

	JS_Tot	BFQE	BFQE_1	BFQE_2	BFQA	BFQA_1	BFQA_2	BFQC	BFQC_1	BFQC_2	BFQS	BFQS_1	BFQS_2	BFQO	BFQO_1	BFQO_2	*M*	*SD*
JS_Tot	-	.247^**^	.058	.252^**^	.209^**^	.142^*^	.223^**^	.159^*^	.001	.283^**^	.185^**^	.142^*^	.188^**^	-.007	.013	-.028	22.40	5.16
BFQE	.243^**^	-	.247^**^	.058	.252^**^	.209^**^	.142^*^	.223^**^	.159^*^	.001	.283^**^	.185^**^	.142^*^	.188^**^	-.007	.013	77.29	1.30
BFQE_1	.053	.780^**^	-	.435^**^	.296^**^	.310^**^	.213^**^	.047	-.157^*^	.270^**^	.199^**^	.302^**^	.045	.442^**^	.352^**^	.436^**^	41.29	5.51
BFQE_2	.250^**^	.857^**^	.407^**^	-	-.009	.047	-.059	.270^**^	.114	.346^**^	.117	.210^**^	-.009	.308^**^	.317^**^	.222^**^	35.50	6.17
BFQA	.194^**^	.205^**^	.308^**^	-.031	-	.870^**^	.887^**^	.090	-.068	.242^**^	.276^**^	.177^*^	.318^**^	.430^**^	**.371**	.392^**^	79.70	9.71
BFQA_1	.121	.242^**^	.320^**^	.025	.872^**^	-	.545^**^	.118	-.046	.265^**^	.185^**^	.132	.200^**^	.484^**^	.444^**^	.409^**^	41.77	5.40
BFQA_2	.218^**^	.122	.223^**^	-.077	.884^**^	.542^**^	-	.043	-.073	.165^*^	.296^**^	.177^*^	.355^**^	.279^**^	.215^**^	.284^**^	37.93	5.65
BFQC	.151^*^	.263^**^	.055	.257^**^	.104	.136	.049	-	.848^*^	.774^**^	-.029	-.058	.009	.299^**^	.352^**^	.163^*^	83.86	11.21
BFQC_1	-.006	.013	-.148^*^	.106	-.052	-.023	-.067	.849^**^	-	.321^**^	-.137	-.210^**^	-.028	.133	.206^**^	.014	40.28	7.49
BFQC_2	.278^**^	.455^**^	.274^**^	.334^**^	.248^**^	.270^**^	.168^*^	.775^**^	.324^**^	-	.111	.146^*^	.049	.376^**^	.384^**^	.274^**^	43.58	6.27
BFQS	.191^**^	.191^**^	.159^*^	.150^*^	.212^**^	.116	.254^**^	-.051	-.152^*^	.091	-	.897^**^	.883^**^	.117	.162^*^	.036	71.49	13.87
BFQS_1	.150^*^	.287^**^	.244^**^	.244^**^	.105	.052	.131	-.082	-.222^**^	.118	.905^**^	-	.584^**^	.164^*^	.189^**^	.094	37.09	8.20
BFQS_2	.193^**^	.041	.028	.012	.284^**^	.161^*^	.334^**^	-.004	-.039	.040	.880^**^	.593^**^	-	.042	.098	-.034	34.40	7.34
BFQO	-.006	.455^**^	.445^**^	.289^**^	.430^**^	.474^**^	.285^**^	.299^**^	.133	.377^**^	.097	.134	.033	-	.902^**^	.864^**^	83.35	1.63
BFQO_1	.022	.396^**^	.348^**^	.295^**^	.354^**^	.408^**^	.218^**^	.339^**^	.194^**^	.376^**^	.157^*^	.177^*^	.098	.897^**^	-	.563^**^	41.38	6.52
BFQO_2	-.037	.405^**^	.442^**^	.205^**^	.405^**^	.426^**^	.289^**^	.173^*^	.026	.278^**^	.001	.048	-.051	.856^**^	.539^**^	-	41.97	5.58
Age	.069	-.056	-.014	-.045	-.082	-.166^*^	.017	-.065	-.073	-.029	.086	.099	.052	.042	.152^*^	-.098	44.82	1.56

Note

*. Correlation is significant at the .05 level (2-tailed).

**. Correlation is significant at the .01 level (2-tailed). JS_Tot = Job Satisfaction Scale total score; BFQE = Extraversion (Big-Five Questionnaire); BFQE_1 = Dynamism (Big-Five Questionnaire); BFQE_2 = Dominance (Big-Five Questionnaire); BFQA = Agreeableness (Big-Five Questionnaire); BFQA_1 = Cooperativeness (Big-Five Questionnaire); BFQA_2 = Politeness (Big-Five Questionnaire); BFQC = Conscientiousness (Big-Five Questionnaire); BFQC_1 = Scrupulousness (Big-Five Questionnaire); BFQC_2 = Perseverance (Big-Five Questionnaire); BFQS = Emotional Stability (Big-Five Questionnaire); BFQS_1 = Control of the emotions (Big-Five Questionnaire); BFQS_2 = Control of the impulses (Big-Five Questionnaire); BFQO = Openness (Big-Five Questionnaire); BFQO_1 = Openness to culture (Big-Five Questionnaire); BFQO_2 = Openness to experience (Big-Five Questionnaire).

Pearson’s analysis showed significant and positive correlations for Job satisfaction with Extraversion (*r* = .243, *p* < .01) and its subdimension Dominance (*r* = .250, *p* < .01), Agreeableness (*r* = .194, *p* < .01) and its subdimension Politeness (*r* = .218, *p* < .01), Conscientiousness (*r* = .151, *p* < .05) and its subdimension Perseverance (*r* = .278, *p* < .01), and Emotional stability (*r* = .191, *p* < .01) and its subdimensions Control of the emotions (*r* = .150, *p* < .05) and Control of the impulses (*r* = .193, *p* < .01). Job satisfaction did not correlate with Age (*r* = .069). Similar results were found with the partial correlation analysis, controlling for Gender and Age: Job satisfaction was significantly and positively associated with Extraversion (*r* = .247, *p* < .01) and its subdimension Dominance (*r* = .252, *p* < .01), Agreeableness (*r* = .209, *p* < .01) and its subdimensions Cooperativeness (*r* = .142, *p* < .05) and Politeness (*r* = .223, *p* < .01), Conscientiousness (*r* = .159, *p* < .05) and its subdimension Perseverance (*r* = .283, *p* < .01), and Emotional stability (*r* = .185, *p* < .01) and its subdimensions Control of the emotions (*r* = .142, *p* < .05) and Control of the impulses (*r* = .188, *p* < .01).

**Fig 2:**
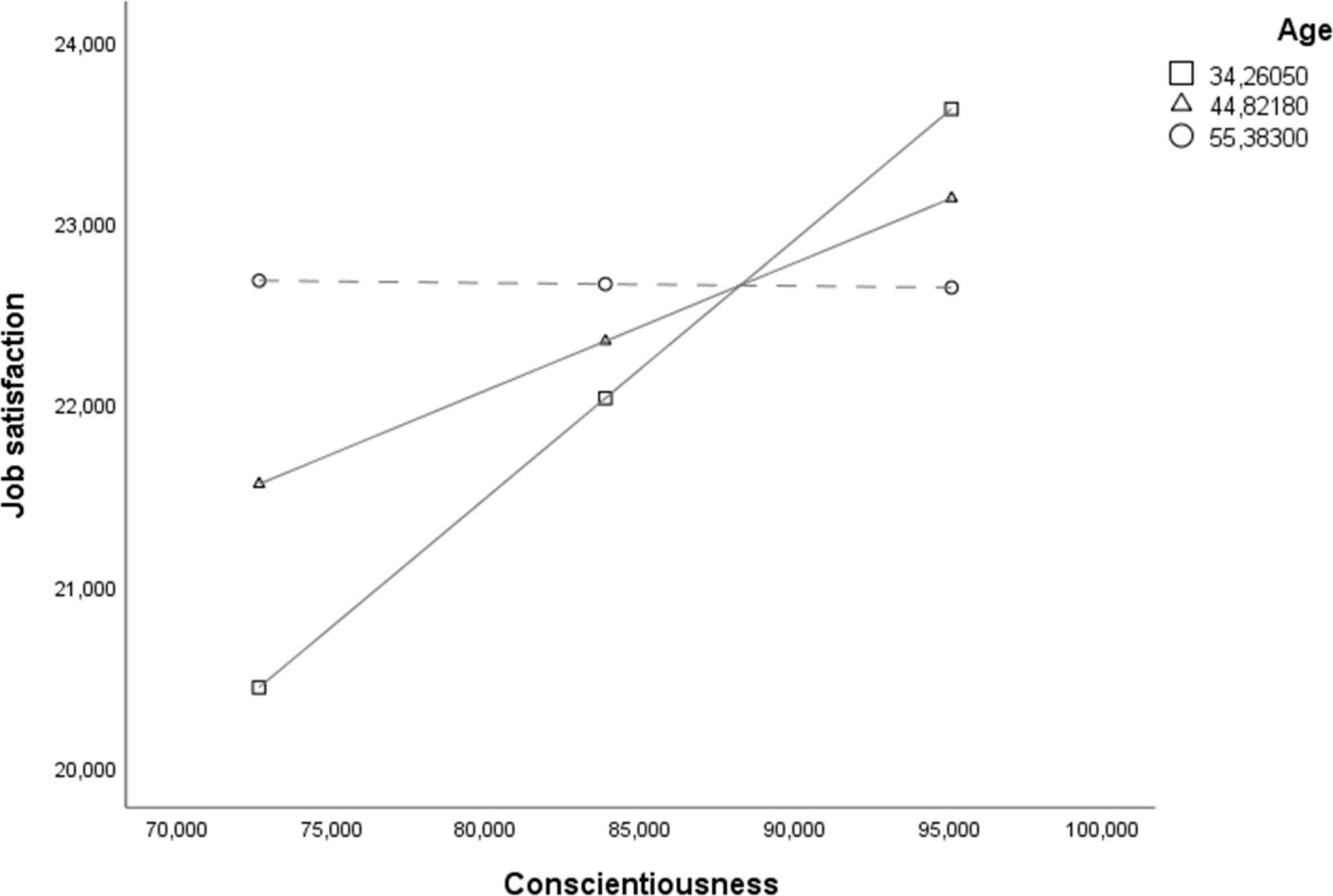
Moderation chart. Graphical representation of the moderation effect.

Concerning the hypothesized moderation model, the results confirmed a significant and positive relationship between Conscientiousness and Job satisfaction (path *b*_*1*_ in Fig 1B; *β* = .82, *p* < .01, LLCI = .093–ULCI = .658), which was moderated by age (path *b*_*3*_ in Fig 1B; *β* = -1.30, *p* < .05, LLCI = -.013–ULCI = -.001): *ΔR*^*2*^ = .023, *F*(1, 198) = 4.724, *p* < .05 (see Table 2, part A). The interaction was probed following Wayne et al. [68] by testing the conditional effects of Conscientiousness at three age ranges (i.e., -1DS, Mean, +1DS). The interaction between Conscientiousness and Job satisfaction was significant, positive, and slightly stronger at low age ranges (estimate = .142(.05), *p* < .01, LLCI = .053–ULCI = .232) than at average age ranges (estimate = .070(.03), *p* < .05, LLCI = .007–ULCI = .133), while it became insignificant at high age ranges (estimate = -.002(.05), *p =* .971, LLCI = -.094–ULCI = .090). Therefore, younger workers showed a more positive association between Conscientiousness and Job satisfaction, which weakened for average-age subjects and became insignificant for older workers (see Fig 2).

**Table 2:** Coefficients of the moderation models.

**A) Model 1: the age-moderated association between Conscientiousness and Job Satisfaction**					
Antecedent	Consequent				
	Y (Job satisfaction)				
		Coeff.	SE	*p*	95% Confidence Intervals
X (Conscientiousness)	*b*_*1*_	.376	.143	.009	[.093; .656]
W (Age)	*b*_*2*_	.601	.261	.022	[.087; 1.116]
Conscientiousness * Age	*b*_*3*_	-.007	.003	.031	[-.013; -.001]
Constant	*i*_*Y*_	-10.494	11.983	.382	[-34,124; 13,136]
	*R*^*2*^ = 0.052 *F*(3, 198) = 3.608, *p =* .014				
**B) Model 2: the age-moderated association between Scrupulousness and Job Satisfaction**					
Antecedent	Consequent				
	Y (Job satisfaction)				
		Coeff.	SE	*p*	95% Confidence Intervals
X (Scrupulousness)	*b*_*1*_	.121	.213	.571	[-.299; .541]
W (Age)	*b*_*2*_	.142	.189	.451	[-.229; .513]
Scrupulousness * Age	*b*_*3*_	-.003	.005	.559	[-.012; .006]
Constant	*i*_*Y*_	16.065	8.662	.065	[-1,016; 3.147]
	*R*^*2*^ = 0.007 *F*(3, 198) = .434, *p =* .729				
**C) Model 3: the age-moderated association between of Perseverance and Job Satisfaction**					
Antecedent	Consequent				
	Y (Job satisfaction)				
		Coeff.	SE	*p*	95% Confidence Intervals
X (Perseverance)	*b*_*1*_	.869	.241	< .001	[.394; 1.344]
W (Age)	*b*_*2*_	.669	.234	.005	[.207; 1.131]
Perseverance * Age	*b*_*3*_	-.015	.005	.007	[-.026; -.004]
Constant	*i*_*Y*_	-16.652	10.435	.112	[-37.230; 3.926]
	*R*^*2*^ = 0.116 *F*(3, 198) = 8.676, *p* < .001				

Furthermore, the significance of the moderation effect was confirmed by the bootstrapping procedure (Boot LLCI = -.013; Boot ULCI = .001). The post-hoc power analysis revealed sufficient power, with a value of 0.80.

Concerning the explorative moderation models, the association between Scrupulousness and Job satisfaction was not significant (*β* = -.01, *p* = .936, LLCI = -.100 –ULCI = .092) and there was not a significant moderation effect (see Table 2, part B). On the other hand, a significant and positive relationship between Perseverance and Job satisfaction was found (*β* = 1.06, *p* < .001, LLCI = .394–ULCI = 1.344), which was moderated by age (*β* = -1.50, *p* < .01, LLCI = -.026–ULCI = -.004): *ΔR*^*2*^ = .033, *F*(1, 198) = 7.407, *p* < .01 (see Table 2, part C). Therefore, the conditional effects of Perseverance at three age ranges (i.e., -1DS, Mean, +1DS) was tested following Wayne et al. [68]. The interaction between Perseverance and Job satisfaction was significant, positive, and slightly stronger at low age ranges (estimate = .363(.07), *p* < .01, LLCI = .218–ULCI = .508) than at average age ranges (estimate = .207(.06), *p* < .001, LLCI = .097–ULCI = .317), while it became insignificant at high age ranges (estimate = .051(.09), *p =* .550, LLCI = -.118–ULCI = .221). Therefore, younger workers showed a more positive association between Perseverance and Job satisfaction, which weakened for average-age subjects and became insignificant for older workers. The bootstrapping procedure confirmed the significance of the age-moderated effect between Perseverance on Job satisfaction (Boot LLCI = -.026; Boot ULCI = .004) and a post-hoc power analysis revealed a high power, with a value of 0.99.

Finally, the moderation effect of age in the relationships between different personality dimensions and job satisfaction was tested. The results showed that no significant alternative moderation models were found (see Table 3).

**Table 3:** Alternative models summaries and indices.

Antecedent	Model Summary	Test of highest order unconditional interaction:	Bootstrapping 95% CI for the Moderation Effect
Extraversion	*R*^*2*^ = .069 *F*(3, 198) = 4.861, *p* < .01	*ΔR*^*2*^ = .003 *F*(1, 198) = .594, *p* = .442	[-.0037; .0082]
Agreeableness	*R*^*2*^ = .047 *F*(3, 198) = 3.272, *p* < .05	*ΔR*^*2*^ = .001 *F*(1, 198) = .457, *p* = .500	[-.0096; .0051]
Conscientiousness	*R*^*2*^ = .052 *F*(3, 198) = 3.608, *p* < .05	*ΔR*^*2*^ = .023 *F*(1, 198) = 4.724, *p* < .05	[-.0132; -.0010]
Emotional Stability	*R*^*2*^ = .039 *F*(3, 198) = 2.700, *p* < .05	*ΔR*^*2*^ = .000 *F*(1, 198) = .000, *p* = .987	[-.0054; .0062]
Openness	*R*^*2*^ = .007 *F*(3, 198) = .460, *p* = .711	*ΔR*^*2*^ = .002 *F*(1, 198) = .406, *p* = .525	[-.0088; .0044]

***Note***: Extraversion = the association between Extraversion and Job satisfaction, moderated by age; Agreeableness = the association between Agreeableness and Job satisfaction, moderated by age; Conscientiousness = the association between Conscientiousness and Job satisfaction, moderated by age; Emotional Stability = the association between Emotional Stability and Job satisfaction, moderated by age; Openness = the association between Openness and Job satisfaction, moderated by age.

## Discussion

The concept of healthy organizations highlights the strong link between health and productivity [35]. Performance and well-being are seen as two interdependent and necessary aspects for a sustainable and successful business [30,72,73]. Within this framework, job satisfaction assumes a key role, where it is strictly related to personal and organizational results and to the life satisfaction of the workers [74]. Therefore, this study set itself the goal of deepening understanding the relationship between dispositions and job satisfaction by exploring the effects of age and conscientiousness.

Consistent to previous research, results showed a significant relationship between conscientiousness and job satisfaction [50,56]. Analyzing more specifically the dimensions of this personality trait, while for the scrupulousness subdimension no significant association was found, that of perseverance showed a significant interaction with job satisfaction. Indeed, scientific literature suggested that workers having high levels of conscientiousness seem more success-oriented, with behaviors aimed at achieving positive results with higher work efficiency: this will favor higher intrinsic and extrinsic rewards, in turn, providing sources of job satisfaction [50,75]. More specifically, perseverance is negatively associated with counterproductive behavior at work and positively with job performance [76], which in turn is related to being more likely to be satisfied with one’s job [77]. However, our results also highlighted greater complexities in these relationships by showing the moderation by age: specifically, although significant association of conscientiousness or perseverance with job satisfaction were found, they were limited to the younger and average-age workers. This is in line with previous studies [60] and could be read as expressing the strong influence of a success-orientation on younger subjects who are still experiencing both personality and professional identity pathways to maturation [78]. On the other hand, in the course of a lifespan, decreased levels of conscientiousness may occur [79]. This may lead to changes in motivation, which could now be directed towards other factors from which the subject derives satisfaction [80]. Lastly, no age-moderated effects were found in the relationship between the other personality traits and job satisfaction, although the data confirmed the results obtained in previous studies highlighting significant and positive correlations between job satisfaction and traits of extraversion [43,81], agreeableness [56,82], and emotional stability [83,84]. Such findings support the role of dispositions and their relevance in exploring the antecedents of job satisfaction, but in parallel, the moderation of age for conscientiousness corroborate the view of the interactive models, that highlighted the need for integration with other factors, such as situational ones, which could gain greater relevance at certain in certain life phases of some workers [85].

This research has some limitations that would be useful to consider. The first concerns the fact that the research is cross-sectional. To effectively evaluate the trajectories of traits and their association with job satisfaction, it would be valuable to carry out longitudinal studies in the future. Moreover, no data about tenure, position or education have been collected, and no occupational differences were investigated. Future research could deepen the integrative approach by exploring these factors in relation to different kinds of work, occupation, and organization (e.g., public or private) and also job position. Additionally, self-report measures were used to gather the data, with the possibility that biases were present. Multimethod-multimodal approaches (e.g., integrating the use of structured or semi-structured interviews) could help to overcome this issue in future studies. Finally, Although the bootstrap technique and post-hoc power analysis supported the statistical stability of the models, it will be necessary further research to replicate and extend results, also integrating information of other sources (e.g., qualitative information and replication studies in other workers samples and different job environments).

## Conclusions

This research provides additional elements to support a better understanding of factors associated with job satisfaction. Specifically, associations between conscientiousness traits and job satisfaction were highlighted, and also the need to integrate the study of dispositional factors with the characteristics of work and situational elements in order to provide a more complete picture of the phenomenon [73,85]. This may have important implications both from a theoretical and applicative point of view. Indeed, such findings increases evidences in line with an integrated approach and stimulates the deepening of both personality and organizational factors in subsequent research. Furthermore, these results could have important applicative implications for the framework of healthy organizations [30,31] and the well-being movement [32,33]. More effective interventions could be planned by suggesting the need for differentiation according to workers’ traits and ages.

## References

[1] Judge TA, Zhang SC, Glerum DR. Job satisfaction. In: Sessa VI, Bowling NA, editors. Essentials of Job Attitudes and Other Workplace Psychological Constructs. Routledge; 2020. pp. 207–241.

[2] Timothy AJ, Klinger R. Job Satisfaction: Subjective well-being at work. In: Eid M, Larsen RJ, editors. The science of subjective well-being. New York: The Guilford Press; 2008. p. 393–413.

[3] Judge TA, Kammeyer-Mueller JD. Job attitudes. Annu. Rev. Psychol. 2012;63: 341–367. doi: 10.1146/annurev-psych-120710-100511 22129457

[4] Srivastava S. Job satisfaction and organizational commitment relationship: Effect of personality variables. Vision. 2013;17(2): 159–167. doi: 10.1177/0972262912483529

[5] Ćulibrk J, Delić M, Mitrović S, Ćulibrk D. Job satisfaction, organizational commitment and job involvement: The mediating role of job involvement. Front. Psychol. 2013;9: 132. doi: 10.3389/fpsyg.2018.00132 29503623 PMC5820360

[6] Judge TA, Scott BA, Ilies R. Hostility, job attitudes, and workplace deviance: Test of a multilevel model. J. Appl. Psychol. 2006;91(1), 126–138. doi: 10.1037/0021-9010.91.1.126 16435943

[7] Sesen H, Basim NH. Impact of satisfaction and commitment on teachers’ organizational citizenship. Educ. Psychol. 2012;32(4): 475–491.

[8] Gillet N, Fouquereau E, Coillot H, Cougot B, Moret L, Dupont S, et al. The effects of work factors on nurses’ job satisfaction, quality of care and turnover intentions in oncology. J. Adv. Nurs. 2018;74: 1208–1219. doi: 10.1111/jan.13524 29350770

[9] Schaumberg RL, Flynn FJ. Clarifying the link between job satisfaction and absenteeism: The role of guilt proneness. J. Appl. Psychol., 2017;102(6); 982–992. doi: 10.1037/apl0000208 28277721

[10] Mount M, Ilies R, Johnson E. Relationship of personality traits and counterproductive work behaviors: The mediating effects of job satisfaction. Pers. Psychol. 2018;59(3): 591–622. doi: 10.1111/j.1744-6570.2006.00048.x</underline>

[11] Czarnota-Bojarska J. Counterproductive work behavior and job satisfaction: A surprisingly rocky relationship. J. Manag. Organ. 2015;21(4): 460–470. doi: 10.1017/jmo.2015.15

[12] Fisher C. Conceptualizing and measuring wellbeing at work. Wellbeing 2014;3: 1–25. doi: 10.1002/9781118539415.wbwell018

[13] Steel P, Schmidt J, Bosco F, Uggerslev K. The effects of personality on job satisfaction and life satisfaction: A meta-analytic investigation accounting for bandwidth–fidelity and commensurability. Hum. Relat. 2019;72(2): 217–247. doi: 10.</underline>1177%2F0018726718771465

[14] Gori A, Topino E, Di Fabio A. The protective role of life satisfaction, coping strategies and defense mechanisms on perceived stress due to COVID-19 emergency: A chained mediation model. Plos one, 2020;15(11): e0242402. doi: 10.1371/journal.pone.0242402 33186367 PMC7665746

[15] Amati M, Tomasetti M, Ciuccarelli M, Mariotti L, Tarquini LM, Bracci M et al. Relationship of job satisfaction, psychological distress and stress‐related biological parameters among healthy nurses: a longitudinal study. J. Occup. Health. 2010;52(1): 31–38. doi: 10.1539/joh.l9042 20032591

[16] Maslach C, Schaufeli WB, Leiter MP. Job burnout. Annu. Rev. Psychol. 2001;52(1): 397–422. doi: 10.1146/annurev.psych.52.1.397 11148311

[17] Kim WH, Ra YA, Park JG, Kwon B. Role of burnout on job level, job satisfaction, and task performance. Leadersh. Organ. Dev. J. 2017;38: 630–645. doi: 10.1108/LODJ-11-2015-0249

[18] Faragher EB, Cass M, Cooper CL. The Relationship between Job Satisfaction and Health: A Meta-Analysis. In Cooper C, editor. From stress to wellbeing volume 1: the theory and research on occupational stress and wellbeing. Berlin, Germany: Springer Science and Business Media LLC; 2013. pp. 254–271.

[19] Wang M. Profiling retirees in the retirement transition and adjustment process: Examining the longitudinal change patterns of retirees’ psychological well-being. J. Appl. Psychol. 2007;92(2): 455–474. doi: 10.1037/0021-9010.92.2.455 17371091

[20] Ashkzari MK, Piryaei S, Brojerdian N, Ashkezari EK. The relationship between job satisfaction with marital satisfaction and mental health: The specific case of female employees. Eur. Psychiatry. 2017;41(S1): S737–S737. doi: 10.1016/j.eurpsy.2017.01.1353

[21] Martínez-Martí ML Ruch W. The relationship between orientations to happiness and job satisfaction one year later in a representative sample of employees in Switzerland. J. Happiness Stud. 2017;18(1): 1–15. doi: 10.1007/s10902-016-9714-4

[22] Lim S, Cortina LM, Magley VJ. Personal and workgroup incivility: Impact on work and health outcomes. J. Appl. Psychol. 2008;93(1): 95–107. doi: 10.1037/0021-9010.93.1.95 18211138

[23] Miah M. The impact of employee job satisfaction toward organizational performance: A study of private sector employees in Kuching, East Malaysia. Int. J. Sci. Res. Publ. 2018;8: 270–278. doi: 10.29322/ijsrp.8.12.2018.p8437</underline>

[24] Blustein DL. The Psychology of Working: A New Perspective for Career Development, Counseling and Public Policy. New York, NY: Routledge; 2006.

[25] Blustein DL. The Oxford Handbook of the Psychology of Working. Oxford: Oxford University Press; 2013.

[26] Blustein DL, Kenny ME, Di Fabio A, Guichard J. Expanding the impact of the psychology of working: Engaging psychology in the struggle for decent work and human rights. J. Career Assess. 2019;27: 3–28; doi: 10.1177/1069072718774002

[27] Duffy RD, Blustein DL, Diemer MA, Autin KL. The psychology of working theory. J. Couns. Psychol. 2016;63(2): 127–148. doi: 10.1037/cou0000140 26937788

[28] Di Fabio A, Blustein DL. From meaning of working to meaningful lives: The challenges of expanding decent work. Front. Psychol. 2016;7: 1119. doi: 10.3389/fpsyg.2016.01119 27512380 PMC4961706

[29] Lowe GS. Creating healthy organizations: How vibrant workplaces inspire employees to achieve sustainable success. University of Toronto Press; 2010.

[30] Di Fabio A. Positive Healthy Organizations: Promoting well-being, meaningfulness, and sustainability in organizations. Front. Psychol. 2017;8: 1938. doi: 10.3389/fpsyg.2017.01938 29184517 PMC5694454

[31] Di Fabio A, Cheung F, Peiró J-M. Editorial Special Issue Personality and individual differences and healthy organizations. Pers. Individ. Differ. 2020;166. doi: 10.1016/j.paid.2020.110196 32834277 PMC7319605

[32] Robertson I, Cooper CL. Wellbeing: Productivity and Happiness at Work. London: Palgrave Macmillan; 2010.

[33] Johnson S, Robertson I, Cooper CL. *Wellbeing*: *Productivity and Happiness at Work*. (2nd ed.). London: Palgrave Macmillan; 2019.

[34] Di Fabio A, Saklofske DH. The relationship of compassion and self-compassion with personality and emotional intelligence in organizations. Pers. Individ. Differ. 2020;169: 110109. doi: 10.1016/j.paid.2020.110109 32394994 PMC7211602

[35] Kivimäki M, Lindström K. Psychosocial approach to occupational health. In: Salvendy G. editor. Handbook of human factors and ergonomics. New York: Wiley; 2006. Pp 801–817.

[36] Di Fabio A, Kenny ME. From decent work to decent lives: Positive Self and Relational Management (PS&RM) in the twenty-first century. Front. Psychol. 2016;7: 361. doi: 10.3389/fpsyg.2016.00361 27047406 PMC4804222

[37] Tetrick LE, Peiró JM. Occupational safety and health. In: Kozlowski SWEditor. The Oxford Handbook of Organizational Psychology, Volume 1. Oxford University Press; 2012.

[38] Hofmann DA, Tetrick LE. On the etiology of health: implications for “organizing” individual and organizational health. In: Hofmann DA, Tetrick LE editors. Health and Safety in Organizations: A Multilevel Perspective. San Francisco, CA: Jossey-Bass; 2003. pp 1–26.

[39] Christensen M. Healthy Individuals in Healthy Organizations: The Happy Productive Worker Hypothesis. In: Christensen M, Saksvik PØ, Karanika-Murray M editors. The positive side of occupational health psychology. Springer, Cham; 2017. pp. 155–169.

[40] Schulte P, Vainio H. Well-being at work–overview and perspective. Scand. J. Work Environ. Health, 2010;36(5):422–429. doi: 10.5271/sjweh.3076 20686738

[41] Gori A, Topino E. Predisposition to Change Is Linked to Job Satisfaction: Assessing the Mediation Roles of Workplace Relation Civility and Insight. Int. J. Environ. Res. Public Health. 2020;17(6): 2141. doi: 10.3390/ijerph17062141 32210195 PMC7143367

[42] Gori A., Topino E., Palazzeschi L., & Di Fabio A. (2020). How Can Organizational Justice Contribute to Job Satisfaction? A Chained Mediation Model. *Sustainability*, 12(19), 7902. 10.3390/su12197902.

[43] Hsieh JY. Impact of individual and organizational factors on job satisfaction: A comparison of multilevel models and multiple regression models using different data arrangements. J. Manag. Organ. 2013;19(1): 44–59. doi: 10.1017/jmo.2013.3

[44] Judge TA, Heller D, Mount M. Five-factor model of personality and job satisfaction: A meta-analysis. J Appl Psychol. 2002;87(3):530–41. doi: 10.1037/0021-9010.87.3.530 12090610

[45] Costa PT Jr, McCrae RR. Personality disorders and the five-factor model of personality. J. Pers. Disord. 1990;4(4): 362–371. doi: 10.1521/pedi.1990.4.4.362</underline>

[46] John OP, Srivastava S. The Big Five trait taxonomy: History, measurement, and theoretical perspectives. In: Pervin LA, John OP editors. Handbook of Personality. New York: The Guilford Press; 1999. pp. 102–138.

[47] Caprara GV, Barbaranelli C, Borgogni L. BFQ: Big Five Questionnaire, 2nd ed. Firenze, Italy; Giunti O.S.; 1993.

[48] Erdheim J, Wang M, Zickar MJ. Linking the Big Five personality constructs to organizational commitment. Pers. Individ. Differ. 2006;41(5): 959–970. doi: 10.1016/j.paid.2006.04.005</underline>

[49] Rubenstein AL, Zhang Y, Ma K, Morrison HM, Jorgensen DF. (2019). Trait expression through perceived job characteristics: A meta-analytic path model linking personality and job attitudes. J. Vocat. Behav. 2019;112: 141–157. doi: 10.1016/j.jvb.2019.02.002

[50] Furnham A, Eracleous A, Chamorro-Premuzic T. Personality, motivation and job satisfaction: Hertzberg meets the Big Five. J. Manag. Psychol. 2009;24(8): 765–779. doi: 10.1108/02683940910996789</underline>

[51] Shaffer JA, Postlethwaite BE. A matter of context: A meta-analytic investigation of the relative validity of contextualized and noncontextualized personality measures. Pers. Psychol. 2012;65(3): 445–93. doi: 10.1111/j.1744-6570.2012.01250.x

[52] Shaffer JA, Postlethwaite BE. The validity of conscientiousness for predicting job performance: A meta-analytic test of two hypotheses. Int. J. Sel. Assess. 2013;21: 183–199. doi: 10.1111/ijsa.12028

[53] Huang JL, Cropanzano R, Li A, Shao P, Zhang XA, Li Y. (2017). Employee conscientiousness, agreeableness, and supervisor justice rule compliance: A three-study investigation. J. Appl. Psychol. 2017;102(11): 1564–1589. doi: 10.1037/apl0000248 28749156

[54] Gartland N, O’Connor DB, Lawton R. The effects of conscientiousness on the appraisals of daily stressors. Stress Health. 2012;28(1): 80–86. doi: 10.1002/smi.1404 22259161

[55] Lapierre LM, Hackett RD. Trait conscientiousness, leader-member exchange, job satisfaction and organizational citizenship behaviour: A test of an integrative model. J. Occup. Organ. Psychol. 2007;80(3): 539–554. doi: 10.1348/096317906X154892</underline>

[56] Bruk-Lee V, Khoury HA, Nixon AE, Goh A, Spector PE. Replicating and extending past personality/job satisfaction meta-analyses. Hum. Perform. 2009;22(2): 156–189.

[57] Miao C, Humphrey RH, Qian S. Leader emotional intelligence and subordinate job satisfaction: A meta-analysis of main, mediator, and moderator effects. Pers. Individ. Dif. 2016;102: 13–24. doi: 10.1016/j.paid.2016.06.056

[58] Costa PT, McCrae RR. Normal personality assessment in clinical practice: The NEO Personality Inventory. Psychol. Assess. 1992;4(1): 5–13. doi: 10.1037/1040-3590.4.1.5

[59] Zhao H, Seibert SE. The big five personality dimensions and entrepreneurial status: A meta-analytical review. J. Appl. Psychol. 2006;91(2): 259–271. doi: 10.1037/0021-9010.91.2.259 16551182

[60] Bui HT. Big Five personality traits and job satisfaction: Evidence from a national sample. J. Gen. Manag. 2017;42(3): 21–30. doi: 10.1177%2F0306307016687990

[61] Baltes BB, Rudolph CW, Bal AC. A review of aging theories and modern work perspectives. In: Hedge JW, Borman WC editors. The Oxford handbook of work and aging. New York: Oxford University Press; 2012. pp. 117–136.

[62] Ebner NC, Freund AM, Baltes PB. Developmental changes in personal goal orientation from young to late adulthood: From striving for gains to maintenance and prevention of losses. Psychol. Aging. 2006;21: 664–678. doi: 10.1037/0882-7974.21.4.664 17201488

[63] Freund AM. Age-differential motivational consequences of optimization versus compensation focus in younger and older adults. Psychol. Aging. 2006;21: 240–252. doi: 10.1037/0882-7974.21.2.240 16768572

[64] Caprara GV, Barbaranelli C, Borgogni L, Perugini M. The “Big Five Questionnaire”: A new questionnaire to assess the five factor model. Pers. Individ. Differ. 1993;15(3): 281–288.

[65] Judge TA, Locke EA, Durham CC, Kluger AN. Dispositional effects on job and life satisfaction: The role of core evaluations. J. Appl. Psychol. 1998;83:17–34. doi: 10.1037/0021-9010.83.1.17 9494439

[66] Di Fabio A. Job Satisfaction Scale: Primo Contributo alla validazione della Versione Italiana [Job Satisfaction Scale: First Contribution to the Validation of the Italian Version]. G. Ital. Ric. Appl. 2018;11. doi: 10.14605/CS1121807

[67] Hayes A.F. Introduction to Mediation, Moderation, and Conditional Process. Analysis: A Regression-Based Approach, 2nd ed.; New York, NY: Guilford Press; 2018.

[68] Wayne SJ, Lemmon G, Hoobler JM, Cheung GW, Wilson MS. The ripple effect: A spillover model of the detrimental impact of work–family conflict on job success. J. Organ. Behav. 2017;38(6): 876–894. doi: 10.1002/job.2174</underline>

[69] Faul F, Erdfelder E, Lang AG, Buchner A. G*Power 3: A flexible statistical power analysis program for the social, behavioral, and biomedical sciences. Behav. Res. Methods. 2007;39:175–191. doi: 10.3758/bf03193146 17695343

[70] Cohen J. *Statistical power analysis for the behavioral sciences*. Academic press; 2013.

[71] Memon MA, Cheah JH, Ramayah T, Ting H, Chuah F, Cham TH. Moderation analysis: issues and guidelines. JASEM. 2019;3(1):1–11. doi: 10.47263/JASEM.3(1)01

[72] De Smet A, Loch M, Schaninger B. Anatomy of a healthy corporation. Mckinsey Q. 2007;2: 64–73.

[73] Peiró JM, Bayonab JA, Caballer A, Di Fabio A. Importance of work characteristics affects job performance: The mediating role of individual dispositions on the work design-performance relationships. PAID 40^th^ Anniversary Special Issue. Pers. Individ. Differ. 2020;157. doi: 10.1016/j.paid.2019.109808

[74] Cohrs JC, Abele AE, Dette DE. Integrating situational and dispositional determinants of job satisfaction: Findings from three samples of professionals. J. Psychol., 2006;140(4): 363–395. doi: 10.3200/JRLP.140.4.363-395 16967742

[75] Templer KJ. Five-factor model of personality and job satisfaction: The importance of agreeableness in a tight and collectivistic Asian society. Appl. Psychol. 2012;61(1): 114–129. doi: 10.1111/j.1464-0597.2011.00459.x

[76] Littman-Ovadia H, Lavy S. Going the extra mile: Perseverance as a key character strength at work. J. Career Assess. 2016;24(2): 240–252. doi: 10.1177%2F1069072715580322

[77] Reed AJ, Schmitz D, Baker E, Nukui A, Epperly T. Association of “grit” and satisfaction in rural and nonrural doctors. JABPEJ. 201225(6), 832–839. doi: 10.3122/jabfm.2012.06.110044 23136323

[78] Alessandri G, Truxillo DM, Tisak J, Fagnani C, Borgogni L. Within-Individual Age-Related Trends, Cycles, and Event-Driven Changes in Job Performance: a Career-Span Perspective. J. Bus. Psychol. 2019; 35: 643–662.

[79] Lucas RE, Donnellan MB. (2011). Personality development across the life span: Longitudinal analyses with a national sample from Germany. J. Pers. Soc. Psychol. 2011;101(4): 847–861. doi: 10.1037/a0024298 21707197

[80] Barrick MR, Mount MK, Li N. The theory of purposeful work behavior: The role of personality, higher-order goals, and job characteristics. Acad. Manage Rev. 2013;38(1): 132–153. doi: 10.5465/amr.2010.0479

[81] Harari MB, Thompson AH, Viswesvaran C. Extraversion and job satisfaction: The role of trait bandwidth and the moderating effect of status goal attainment. Pers. Individ. Differ. 2018;123: 14–16. doi: 10.1016/J.PAID.2017.10.041

[82] Ilies R, Fulmer IS, Spitzmuller M, Johnson MD. Personality and citizenship behavior: The mediating role of job satisfaction. J. Appl. Psychol., 2009;94(4): 945. doi: 10.1037/a0013329 19594236

[83] Ilies R, Judge TA. On the heritability of job satisfaction: The mediating role of personality. J. Appl. Psychol. 2003;88(4): 750–759. doi: 10.1037/0021-9010.88.4.750 12940413

[84] Judge TA, Heller D, Klinger R. The dispositional sources of job satisfaction: A comparative test. Appl. Psychol. 2008;57(3): 361–372. doi: 10.1111/j.1464-0597.2007.00318.x

[85] Judge TA, Parker SK, Colbert AE, Heller D, Ilies R. Job satisfaction: A cross-cultural review. In: Anderson N, Ones DS, Sinangil HK, Viswesvaran C, editors. Handbook of industrial, work and organizational psychology, Vol. 2. Organizational psychology Sage Publications, Inc; 2002. pp 25–52.

